# Extended visuomotor experience with inverted movements can overcome the inversion effect in biological motion perception

**DOI:** 10.1038/s41598-022-21000-1

**Published:** 2022-10-20

**Authors:** Xiaoye Michael Wang, Margaret A. Wilson, Yu Song, Gulandanmu Ma, Mingkai Dong, Qin Zhu

**Affiliations:** 1 Department of Kinesiology, Faculty of Kinesiology and Physical Education, University of Toronto, Toronto, ON Canada; 2 Department of Theatre and Dance, University of Wyoming, Laramie, WY USA; 3 Division of Kinesiology and Health, University of Wyoming, Laramie, WY USA; 4 Department of Sports Choreography, Shanghai University of Sport, Shanghai, China; 5 Department of Physical Education, Shanghai University of Finance and Economics, Shanghai, China

**Keywords:** Psychology, Human behaviour

## Abstract

Studies have demonstrated that perceiving human and animal movements as point-light displays is effortless. However, simply inverting the display can significantly impair this ability. Compared to non-dancers and typical dancers, vertical dancers have the unique experience of observing and performing movements upside down as being suspended in the air. We studied whether this unique visuomotor experience makes them better at perceiving the inverted movements. We presented ten pairs of dance movements as point-light displays. Each pair included a version performed on the ground whereas the other was in the air. We inverted the display in half of the trials and asked vertical dancers, typical dancers, and non-dancers about whether the display was inverted. We found that only vertical dancers, who have extended visual and motor experience with the configural and dynamic information of the movements, could identify the inversion of movements performed in the air. Neither typical dancers nor non-dancers, who have no motor experience with performing the inverted movements, could detect the inversion. Our findings suggest that motor experience plays a more critical role in enabling the observers to use dynamic information for identifying artificial inversion in biological motion.

Biological motion perception has been widely studied since Gunnar Johansson^1,2^ presented the first series of point-light displays (PLD). Originally, Johansson placed a series of reflective tapes onto the major joints of an actor and recorded various movements. The resulting display contains ten bright points presented against a black background. Only using the apparent motion of these points, studies have established that human observers were able to recognize the type of action being performed^3^, the actors’ gender^4^, and even identity^5^.

In the study of biological motion perception, there has been a debate on how to account for this ability to extract shape and dynamic event information from a few moving points (see^6^ for a review). On the one hand, some studies suggested that observers first extract the static structural information of the object in the display and use this information to derive the dynamic information that subsequently facilitates event recognition (*configural processing*^7–9^). For instance, Beintema and Lappe^7^ placed the points on a walker’s limbs instead of the joints, and randomly varied the points’ locations on the limb for each frame. The authors argued that this manipulation retained the configural information of the walker while eliminating the dynamic information of each point as compared to the conventional PLD. Based on their findings that suggested observers could still discriminate the walker’s walking directions despite the perturbation, the authors argued that biological motion processing should start with sequences of static postures, which are then integrated over time to yield information about the event. On the other hand, the ability to perceive biological motion could just rely on the dynamic information in the display (*dynamic processing*^10^). According to the *kinematic specification of dynamics* theory^10^, the movements of an object (kinematics) are dictated by the underlying forces acting on the object (dynamics). In the context of a PLD walker, the entire force environment that the walker is in, such as the gravitational force, determines the motions of walking limbs as captured through the point lights. The dynamic information, in turn, specifies various aspects of the event (e.g., walking direction^11^) and the object (e.g., gender of the walker^12^).

In addition to the configural and dynamic information, biological motion perception also relies on the observers’ ability to recognize the actions, which, in turn, is contingent upon the observers’ visuomotor experience (see^13^ for a review). A major debate in this area concerns the relative importance of visual and motor experience. For instance, Calvo-Merino and colleagues^14,15^ studied biological motion perception among professional ballet dancers. In ballet, male and female dancers are commonly trained to perform different movements, resulting in different levels of motor experience. However, dancers of both genders would routinely train and perform together, indicating an equal level of visual experience of movements performed by both genders. Only using ballet movements performed by a female dancer, Calvo-Merino et al.^15^ asked male and female ballet dancers, as well as non-dancers, whether the two movements were identical. They found that both male and female dancers were more sensitive to different ballet movements as compared to non-dancers, indicating the importance of visual experience. They also found that female dancers were slightly more sensitive than male dancers, which indicates the additional contribution of motor experience to biological motion perception.

Finally, there is also an interaction between the information of biological motion and the observers’ visuomotor experience. Despite the robustness of biological motion perception, studies revealed that inverting the PLD from its canonical orientation would impair the observers’ ability to recognize the movement (the inversion effect of biological motion^16,17^). Based on the *configural processing theory*, the inversion effect is attributed to the inversion of the spatial layout of a person, presenting the person’s posture in an unfamiliar orientation and rendering it difficult to recognize. Alternatively, based on the *dynamic processing theory*, this should be attributed to the unfamiliar dynamic relations specified by the kinematics, such as that the direction of gravity is inverted. To examine the reason behind the inversion effect, Shipley^18^ presented a point-light display of a person walking on either his hands or feet and compared the movement detection threshold when the display was either upright or inverted. When the walker walked on his hands and the PLD was upright, the display contained familiar dynamic information but unfamiliar configural information. Conversely, when the display was inverted, it contained unfamiliar dynamic information but familiar configural information. Results confirmed that observers were more sensitive to movements when the display was upright than when it was inverted, implying that the dynamic information should be accountable for the inversion effect in biological motion perception.

Given the interesting finding from Shipley^18^ concerning the manipulation of observers’ familiarity with the configural and dynamic information in the PLD, the current study aims to take this approach a step further by studying the biological motion perception of vertical dance. Vertical dance is a type of aerial modern dance that requires a dancer to be suspended in a harness and perform sequences of movements while in the air. Compared to ground-based dance, vertical dance is constrained by the harness and the rope when the dancer is not interacting with a wall: Dancers’ movements will result in additional translation and swinging movements that need to be controlled. Therefore, compared to their ground counterpart, vertical dancers had a unique experience in dealing with the additional pendulum dynamics that people without vertical dance experiences would not have. Additionally, hanging in the air also allows vertical dancers to perform movements in either an upright or upside-down orientation. When a vertical dancer performs a movement in an upright orientation in the air, the resulting configural information would be identical to that performed on the ground. When the movement is performed in an inverted orientation, the configural information would be different and unfamiliar to those without vertical dance experiences. Alternatively, the opposite would be true if the display itself was inverted. Finally, performing a movement upside down also introduces perturbations to the display’s dynamic information: The relationship between the movement’s orientation and the direction of gravity is flipped when compared to the same movement performed in an upright orientation.

In this study, we specifically examined how information (configural and dynamic) and visuomotor experience would affect biological motion perception of vertical dance among vertical dancers (VD), typical dancers (TD), and non-dancers (ND). We recorded two trained vertical dancers performing a series of dance movements on the ground and in the air and presented the movements as PLDs. Each pair of air and ground movements had an identical form. For half of the pairs, the air and ground movements also had an identical orientation (e.g., both are upright; congruent), whereas, for the other half, the movements had opposite orientations (e.g., ground movement is upright whereas air movement is upside down, or vice versa; incongruent). In other words, the canonical orientation of the congruent air movements was identical to their ground counterparts, whereas that of the incongruent air movements was the opposite. To manipulate the information concerning the dynamic information, we first edited the duration of the PLDs so that one set of displays shows the entire movement sequence (complete) while the other set only displays the final form achieved by the dancer (limited). Then, we turned the PLDs upside-down and mixed them with the originals before asking participants to observe and determine whether the display was presented as-is or artificially inverted.

Regarding the amount of dynamic information, if configural information is critical to biological motion perception, then sensitivity to the artificial inversion in the complete and limited displays should be equivalent. Alternatively, if dynamic information is more important, performance should be worse in the limited display. As for visual experience, all three participant groups could be familiar with ground movements in terms of the dynamic information. Therefore, if observers rely on dynamic information to perceive biological motion, we should expect equivalent performance among all participants with the ground movements.

The key interest of this experiment is the analysis of the air movements. Because of their extended visuomotor experience with both upright and inverted movements, VDs should be sensitive to the artificial inversion of the air movements as well as that of their ground counterparts in both congruent and incongruent conditions. As for TDs and NDs, their lack of visuomotor experience with movements performed in the air which have an opposite canonical orientation to the ground movements (i.e., incongruent air movements) would limit their ability to use the inverted configural and dynamic information to identify inverted movement. Due to their extended visuomotor experience with the ground movements, they would be more likely to judge the incongruent air movements as the inverted ground movements.

## Methods

### Participants

52 adults volunteered in this study. There were 15 participants without any dance experience (Non-Dancers/ND), 21 participants with an average of 7.71 years (SD = 3.62) of typical dance experience (Typical Dancers/TD), and 16 participants with 4.75 years (SD = 2.29) of vertical dance experience (Vertical Dancers/VD). Other personal information was not collected due to privacy concerns. This experiment was performed in accordance with relevant ethical guidelines and regulations of the University of Wyoming Institutional Review Board (IRB) and was approved by the University of Wyoming IRB. All participants provided informed consent prior to participating in the experiment.

### Stimuli and apparatus

We recorded dance movements performed by two professional vertical dancers, a male and a female, using a Vicon Motion Capture System with 8 Bonita Optical cameras at a 160 Hz sampling frequency. We fitted 13 optical markers to each dancer’s major joints, including their left and right hips, knees, ankles, shoulders, elbows, wrists, and the head. Both dancers provided their informed consent for their recorded motion to be used in this experiment.

**Figure 1:**
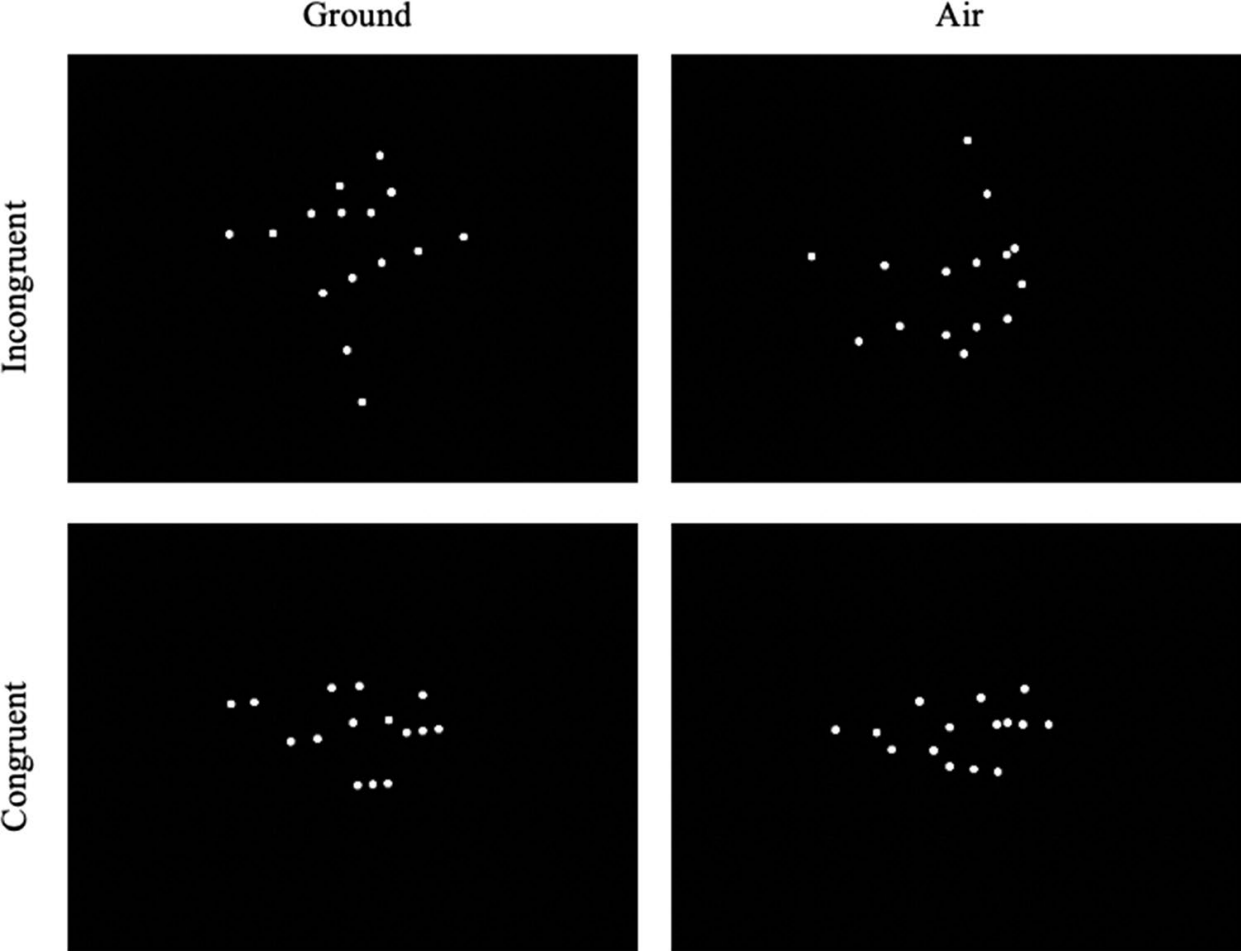
Illustration of the movements of different congruency and recording types. For the incongruent movement pairs (top row), the original (i.e., uninverted) ground and air movements had identical forms but opposite orientations. For the congruent movement pairs (bottom row), the original ground and air movements had identical forms and orientations. See the video demo (https://youtu.be/6vLQgQpy-uc) for more details.

Each dancer performed 10 pairs of dance movements, with one on the ground and the other in the air (Table 1). Each ground-air movement pair had the same form, except that five pairs contained air movements performed in the opposite vertical orientation as their ground counterparts (incongruent condition), whereas for the other five pairs, air and ground movements had identical orientation (congruent condition; Fig. 1). For instance, if a movement was performed upright on the ground with the dancer’s head above the feet, the incongruent version of this movement performed in the air would be upside-down with the dancer’s feet remaining above the head, whereas the congruent version of the air movement would be upright with the dancer’s head above the feet.

**Table 1:** List of dance movements.

Ground	Air	Congruency
Arabesque fondu	Inverted arabesque fondu	Incongruent
Attitude devant over forced arch/arms forward	Inverted attitude devant/arms forward	Incongruent
Développé à la seconde	Inverted développé à la seconde	Incongruent
Italian pas de chat	Inverted pas de chat	Incongruent
X − Plié/extend	Inverted X − Plié/extend	Incongruent
Attitude spiral	Side lying attitude	Congruent
Cambre back B +	High release one leg attitude	Congruent
Plank to V-sit	Plank to V-sit	Congruent
Supported handstand flex/point	Inversion hook foot	Congruent
Side plank—hip lower and lift	Side lying limbs lower/lift	Congruent

Error bars represent standard error of the mean.

We ran the experiment using a custom JSPsych plugin^19^ written in JavaScript hosted on Pavlovia (https://pavlovia.org/). The movements were presented using point-light displays, where each joint was represented by a filled white circle with a radius of 3 pixels against a black background (for a demo, see https://youtu.be/6vLQgQpy-uc). We trimmed different movement sequences to eliminate extraneous movements before and after the target movements and manipulate the amount of available dynamic information in the display. Movements performed by the female dancer were presented in their entirety to provide more dynamic information (complete condition). Each movement sequence had different durations (mean = 4.05 s, SD = 1.10, maximum = 5.94 s, minimum = 1.25 s). Movements performed by the male dancer were more stable and shorter in duration due to the better core stability of the dancer^20^. Therefore, we trimmed each movement performed by the male dancer to its final second, where the dancer has already achieved the final form and remained relatively static, to restrict the amount of dynamic information in the display (limited condition). Finally, we presented the movements either as-is or with each joint’s *y*-coordinate inverted.

### Procedure

Participants accessed the experiment online using a web browser. They were first informed of the study’s general aim, procedure, and data privacy conditions as required by the relevant guidelines issued by the University of Wyoming IRB. The experiment comprised a training phase and two experimental blocks. Each block was followed by a short questionnaire on participants’ strategy and confidence in performing the task. Finally, upon the completion of the experiment, participants provided information regarding their relevant dance experience and the general strategies used to perform the task. The entire experiment took approximately 30 min to complete. Because the experiment was not timed and participants controlled the flow of the experiment themselves, they could take a break between each block.

After providing their informed consent, participants entered the training phase. During training, participants were shown a series of instructions accompanied by a movement (Italian/Inverted Pas de Chat). Animations used during the training phase did not appear in the experimental trials. The instructions stated that participants would see various dance movements, performed either on the ground or in the air. Some movements might be long while others might be short. Some movements might be vertically inverted. For instance, the movement might be recorded with the dancer being in an upright orientation, but it would be displayed in an upside-down orientation. Participants’ task was to determine whether the movement had been artificially inverted by pressing the F (for inverted) or J (for not inverted) on the keyboard. After going through the instructions, participants completed four practice trials and received feedback after each. The practice trials used the same movement presented during the instruction. These movements were recorded either on the ground or in the air and were either presented as-is or artificially inverted.

After the training, the experimental phase started. The experiment was blocked by the amount of dynamic information (limited or complete) and the blocks were presented in a random order for different participants. Each block contained 40 trials (10 movements × 2 recording types [ground or air] × 2 inversion types [as-is or inverted]) in random order. After each block, participants rated their confidence level of judgments on a 5-point Likert scale (1 being the least while 5 being the most confident) and described their strategy to perform the task. Finally, at the end of the experiment, participants quantified years of experience with typical or vertical dancing, ranked which block they found to be harder, and any other general comments or thoughts that they have about the experiment.

### Data analysis

We used two-tailed Wilcoxon signed-rank tests to evaluate participants’ self-reported confidence, comparing (1) confidence of the limited condition with that of the complete conditions and (2) confidence within each category with the neutral level (i.e., 2.5). The Wilcoxon signed-rank test was chosen because of the non-parametric nature of the confidence scale. Then, we used signal detection theory to analyze the judgment data. For each participant, we divided the data based on dynamic types (complete or limited), pair congruency (congruent or incongruent), and recording types (ground or air), resulting in 10 trials per subset of data. For each subset, we considered the signal to be when the display was artificially inverted and, therefore, the signal is absent when the movement was displayed as-is. We calculate the corresponding hit and false alarm rates and derived d-primes (d’) for sensitivity. According to Macmillan and Creelman^21^, a d’ of 0 means that the participants could not distinguish when the display was artificially inverted, whereas a value greater than 1 means that the participants were above the chance level to detect the artificial inversion. Finally, a negative d’ indicates there are more false alarms than hits, and in the current context, represents the case in which an as-is movement was judged to be an artificially inverted movement.

We used a linear mixed model to evaluate the effects of different factors and different dance experiences on d’. The design of this experiment entails one between-subject factor (experience level) and three within-subject factors (dynamic types, pair congruency, and recording types). Due to the complexity of the experimental design and the unequal sample size of different participant groups, we used the linear mixed model (LMM) to analyze the effects of different factors on d’, instead of a conventional analysis of variance (ANOVA). The main benefit of using LMM in the current context is that, unlike ANOVA, it works with unequal samples and allows us to directly compare d’ values among different factor levels without the concern of inflated Type I errors^22,23^. Using the afex^24^ and lme4^25^ packages in R, we designed a statistical model with fixed and random effects:

$$\begin{aligned} & d^{\prime}\sim experience \times dynamic \times congruency \times recording + \left( {1|dynamic:participant} \right) \\ & \quad + \left( {1|congruency:participant} \right) + \left( {1|recording:participant} \right) + (1|participant) \\ \end{aligned}$$

The fixed effect term, $experience\times dynamic\times congruency\times recording$, captures the effects of the between- and within-subject factors and their interactions. We chose ND (experience), limited (dynamic), congruent (congruency), and ground movement (recording type) as baselines. The random effect terms, $\left(1|dynamic:participant\right)+ \left(1|congruency:participant\right)+ \left(1|recording:participant\right)+(1|participant)$, capture the participant-level variability that corresponds to the three within-subject factors. We fitted the model using the restricted maximum likelihood method and estimated degrees of freedom using Kenward-Roger approximation^26^ to compute the *p* values. We used methods proposed by Rights and Sterba^27,28^ to partition and calculate ${R}^{2}$ values for model evaluation using R’s r2mlm package^29^. We will report the proportion of total outcome variance explained by the fixed effects (${R}_{t}^{2\left(f\right)}$) and by the entire model (${R}_{t}^{2\left(fvm\right)}$). Finally, because of the model’s complexity, we performed planned contrasts with Bonferroni correction^30^ using the emmeans package^31^ to explore the source of the significant interaction, comparing d’ across different factor levels.

## Results

Survey results suggested that all three groups of participants were less confident about their judgments in the limited condition (NDs: mean = 1.20, SE = 0.23; TDs: mean = 1.52, SE = 0.19; VDs: mean = 1.44, SE = 0.13) than the complete condition (NDs: mean = 2.00, SE = 0.21, Wilcoxon signed-rank test *W* = 222, *p* < 0.05; TDs: mean = 2.33, SE = 0.20, *W* = 319, *p* < 0.01; VDs: mean = 2.31, *SE* = 0.16, *W* = 194.5, *p* < 0.01). Overall, participants were not confident about performing the task in the limited condition, as confidence for all participant groups was below the neutral level (NDs: *V* = 19, *p* < 0.01; TDs: *V* = 18, *p* < 0.001; VDs: *V* = 6, *p* < 0.001).

**Figure 2:**
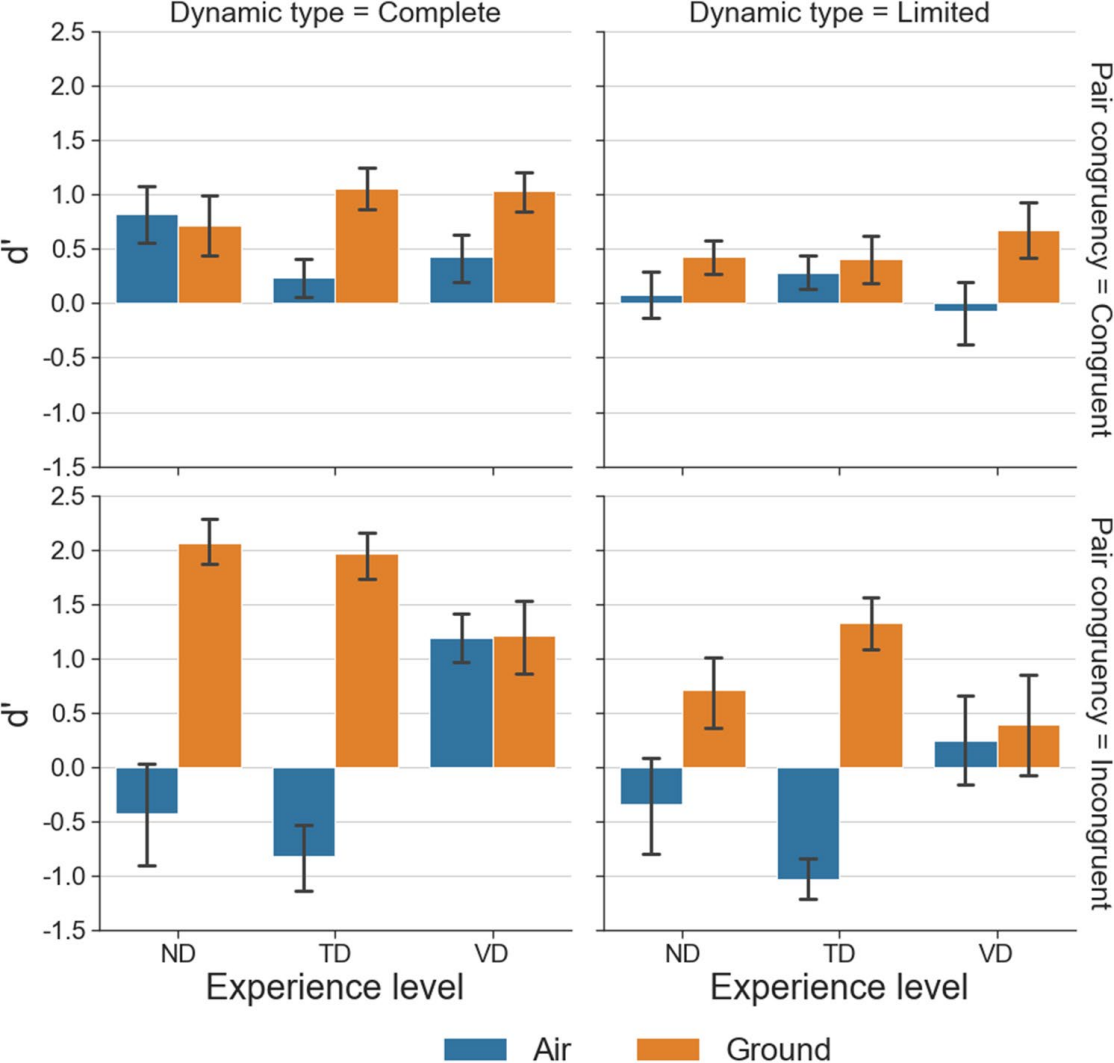
Mean d’ as a function of experience level (*x*-axis; ND: Non-Dancers, TD: Typical Dancers; VD: Vertical Dancers), dynamic type (column), pair congruency (row), and recording type (hue).

Figure 2 illustrates the mean d’ as a function of experience level, dynamic type, pair congruency, and recording type whereas Table 2 summarizes the LMM output. The overall fit of the LMM model was good (${\mathrm{R}}_{\mathrm{t}}^{2\left(f\right)}=0.26,{\mathrm{R}}_{\mathrm{t}}^{2\left(fvm\right)}=0.40$, adjusted ICC = 0.19, $\sigma$ = 1.04, RMSE = 0.95). The model revealed that dynamic type was significant ($F\left(1, 49\right)=26.24, p<0.001, {\eta }_{p}^{2}=0.35$) and its positive slope (0.27 ± 0.05) indicates that the sensitivity in the complete condition was higher than that in the limited condition. This significant and positive effect of dynamic type confirms our hypothesis that the dynamic information is more effective in specifying the inversion in the display than the configural information. Recording type was also significant ($F\left(\mathrm{1,49}\right)=43.67, p<0.001, {\eta }_{p}^{2}=0.47)$ and its negative slope ($-0.47\pm 0.07$) indicates that the sensitivity in the air condition was much lower than that in the ground condition. This significant negative effect supports our hypothesis that participants’ general familiarity with ground-based activities allowed them to be more sensitive to the inversion when the movements were performed on the ground.

**Table 2:** Results of the LMM Model, including the unstandardized ($b$) and standardized ($\beta$) slopes, and t-test results. Experience [ND], Recording Type [Ground], Congruency [Congruent], Dynamic Type [Limited] were used as baselines.s

	$b \left(SE\right)$	$\beta \left(SE\right)$	t	p	Significance
(Intercept)	0.52 (0.07)	0.00 (0.00)	7.29	< 0.001	***
Experience [TD]	− 0.02 (0.10)	− 0.01 (0.06)	− 0.17	0.87	
Experience [VD]	− 0.10 (0.10)	− 0.06 (0.06)	− 0.99	0.32	
Recording type [Air]	− 0.47 (0.07)	− 0.35 (0.05)	− 6.61	< 0.001	***
Congruency [Incongruent]	− 0.02 (0.05)	− 0.01 (0.04)	− 0.33	0.74	
Dynamic type [Complete]	0.27 (0.05)	0.19 (0.04)	− 5.12	< 0.001	***
Experience [TD] × Recording type [Air]	0.00 (0.10)	0.00 (0.06)	0.01	0.99	
Experience [VD] × Recording type [Air]	− 0.29 (0.10)	− 0.18 (0.06)	− 2.97	< 0.01	**
Experience [TD] × Congruency [Incongruent]	0.02 (0.08)	0.01 (0.04)	0.29	0.77	
Experience [VD] × Congruency [Incongruent]	0.08 (0.07)	0.05 (0.04)	1.22	0.22	
Recording Type [Air] × Congruency [Incongruent]	0.26 (0.05)	0.19 (0.04)	5.07	< 0.001	***
Experience [TD] × Dynamic type [Complete]	0.02 (0.08)	0.01 (0.04)	− 0.30	0.76	
Experience [VD] × Dynamic type [Complete]	− 0.08 (0.07)	− 0.05 (0.04)	1.21	0.23	
Recording Type [Air] × Dynamic type [Complete]	− 0.08 (0.05)	− 0.06 (0.04)	1.49	0.14	
Congruency [Incongruent] × Dynamic type [Complete]	− 0.06 (0.05)	− 0.04 (0.04)	1.11	0.27	
Experience [TD] × Recording type [Air] × Congruency [Incongruent]	0.15 (0.08)	0.08 (0.04)	1.98	0.049	*
Experience [VD] × Recording type [Air] × Congruency [Incongruent]	0.26 (0.07)	0.16 (0.04)	3.75	< 0.001	***
Experience [TD] × Recording type [Air] × Dynamic type [Complete]	− 0.05 (0.08)	− 0.03 (0.04)	0.63	0.53	
Experience [VD] × Recording type [Air] × Dynamic type [Complete]	− 0.06 (0.07)	− 0.04 (0.04)	0.91	0.36	
Experience [TD] × Congruency [Incongruent] × Dynamic type [Complete]	0.03 (0.08)	0.02 (0.04)	− 0.38	0.71	
Experience [VD] × Congruency [Incongruent] × Dynamic type [Complete]	0.03 (0.07)	0.02 (0.04)	− 0.38	0.70	
Recording type [Air] × Congruency [Incongruent] × Dynamic type [Complete]	0.07 (0.05)	0.05 (0.04)	− 1.30	0.19	
Experience [TD] × Recording type [Air] × Congruency [Incongruent] × Dynamic type [Complete]	0.17 (0.08)	0.10 (0.04)	− 2.24	0.026	*
Experience [VD] × Recording type [Air] × Congruency [Incongruent] × Dynamic type [Complete]	− 0.10 (0.07)	− 0.06 (0.04)	1.47	0.14	

*$$p<0.05$$, **$$p<0.01$$, ***$$p<0.001$$.

The interaction between experience level and recording type was significant ($F\left(2, 49\right)=5.70, p<0.01, {\eta }_{p}^{2}=0.19$). Regression slopes revealed that the difference in the ground-air differences between TDs and NDs was at around 0 ($0.00\pm 0.10$). As one-tailed planned contrasts suggested, both NDs and TDs were more sensitive to the inversion when the movements were performed on the ground than in the air ($t\left(49\right)=3.58, p<0.001$ for NDs and $t\left(49\right)=6.81, p<0.001$ for TDs). Therefore, the regression slope of around zero suggested that the relative performance in the ground and air conditions were equivalent for TDs and NDs. On the other hand, there was a significant negative regression slope ($- 0.29\pm 0.10$ ) for the ground-air differences between TDs and NDs. Planned contrast showed that VDs were equally sensitive to the air and ground movements ($t\left(49\right)=1.47, p>0.05$), i.e., there was a lack of ground-air difference for the VDs, which supported the effect of the negative regression slope. Overall, this finding supported our hypothesis that VDs’ extended visuomotor experience in vertical dance allowed them to overcome the inversion effect: Unlike NDs and TDs, VDs were equally capable of identifying the artificial inversion regardless of whether the movement was performed on the ground or in the air.

There was also a significant interaction between congruency and recording type ($F\left(1, 196\right)=25.72, p<0.001, {\eta }_{p}^{2}=0.12$) and a significant three-way interaction between experience level, congruency, and recording type ($F\left(2, 196\right)=16.29, p<0.001, {\eta }_{p}^{2}=0.14$). None of the other main or interaction effects were significant. Because the three-way interaction also reflects the two-way interaction between congruency and recording type, we decided to evaluate both effects together using planned contrasts at different levels of congruency and recording type for each experience level. Planned contrasts did not reach significance when comparing sensitivities among NDs, TDs, and VDs for the ground movements when the movements were either congruent ($p>0.9$ for all three pairwise comparisons) or incongruent ($p>0.1$ for all three). Without any prior dance experience, NDs’ performance was still equivalent to that of the TDs and VDs, suggesting that the visual experience of viewing actions, not necessarily dance movements, on the ground is sufficient for detecting the artificial inversion of the dance movements on the ground.

More interesting results emerged from the comparisons between the air and ground movements for different participants and congruency conditions using one-tailed tests. NDs and TDs were both less sensitive to the artificial inversion of the air movements than that of the ground movements when the movements were incongruent (NDs: $t\left(106\right)=-5.43, p<0.001$; TDs: $t\left(106\right)=-9.33, p<0.001$). However, this did not apply to the congruent condition ($p>0.9$ for both). For NDs and TDs, their lack of visuomotor experience with the inverted movements (relative to their ground counterparts) in the air prevented them from using the configural or dynamic information to detect the artificial inversion of those movements. In fact, both NDs and TDs had negative mean d’ values (ND: mean = − 0.39, 95%CI = [− 0.84, 0.07]; TD: mean = − 0.92, 95%CI = [− 1.31, − 0.54]) for the incongruent air movements. A negative d’ suggests that there were more false alarms than hits. In this context, this means that NDs and TDs tended to judge the opposite orientation of air movements (as compared to the ground movements) as the result of artificial inversion. This finding reflects the significant two-way interaction between recording type and congruency. The positive slope ($0.26\pm 0.05$) of the two-way interaction suggests that participants were less sensitive to the artificial inversion with the incongruent air movements.

In contrast, VDs performed equally well with the air movements as they did with the ground movements regardless of the congruency ($p>0.4$ for both). Because of VDs’ extended visuomotor experience with vertical dance, they could pick up the dynamic information in the air movements and use it to judge the inversion in the display, enabling them to be more accurate in judging the artificial inversion for the incongruent air movements compared to the NDs ($t\left(194\right)=1.11, p<0.05$) and TDs ($t\left(194\right)=1.64, p<0.001$). Lastly, comparing VDs with TDs, the former had both visual and motor experience whereas the latter only had visual experience with the configural and/or dynamic information of the movements. TDs’ relatively poor performance with the incongruent air movements suggests that motor experience plays a more critical role in utilizing the dynamic information in the display to perform the judgment.

## Discussion

Biological motion perception is a fascinating ability that seems to even extend beyond humans^32^. Developing an understanding of this ability can shed light on the fundamental mechanisms of visual event perception. The current study combined two core elements of the study of biological motion perception: the underlying visual information responsible for this ability and the mediating effect of the observer’s visuomotor experience.

Regarding the effect of dynamic information, the results of the present study suggested that although observers could use configural information to identify artificial inversion in most cases, the addition of dynamic information improves this ability. Studies have attributed the biological motion inversion effect to the impaired visual processing of either the configural information^16,33^ or the dynamic information^11^. Findings from the current study confirm the importance of dynamic information in biological motion perception and support the kinematic specification of dynamics theory^10,34^. According to this theory, the spatiotemporal patterns of the movement specify not only the intention and identity of the actor, but also the consequence of the movement. Additionally, our finding also extends results from previous studies^11,35^ showing that dynamic information has contributed to perceiving inverted PLDs.

Regarding the role of visuomotor experience, we found that all participant groups were equally sensitive to the inversion when the movements were performed on the ground. However, only VDs, who had the unique experience of observing and performing dance movements upside down when their bodies are suspended in the air through a harness, were sensitive to the artificial inversion when the movements were performed in the air. This finding is in line with previous studies working with professional dancers, where the authors found that both types of visual experiences alone were sufficient for biological motion perception, but the addition of motor experience could further improve this ability^14,15^.

The most critical finding of the current study concerns how participants’ familiarity with the configural and dynamic information affected their ability to discriminate between the actual and artificial inversion, and how this effect is contingent upon the air and ground movements’ congruency. Because of VDs’ visuomotor experience with vertical dance, they were able to utilize the configural and dynamic information in the display and identify the inversion regardless of the movements’ congruency. VD’s ability to detect the inversion of incongruent air movements being as good as that of ground movements suggests that vertical dancers were immune to the inversion effect in biological motion perception. Interestingly, such an ability was also seen for VDs in judging limited motions. In other words, VD’s unique perceptual and motor experience not only remedies the adverse effect of the impaired dynamic information in the display, but also that of the impaired configural information.

In contrast, because TDs only had visual experience with the dance movements performed upright on the ground, they were only able to identify the inversion when the configural information matches what they are familiar with as in the case of congruent air movements. For the incongruent air movements, on the other hand, only the dynamic information is useful in specifying the inversion while the configural information could be rather confusing. Specifically, the inverted incongruent air movements contained the configural information (in an upright orientation) that TDs were familiar with and, because of a lack of visuomotor experience with the inverted movements, they could only rely on the configural information in the display, which has led to poor performance and inflated false alarm in this condition. Finally, although NDs did not have any extended motor or perceptual experience with the dance movements in a professional setting, they are still familiar with the ground-based movements, which contributed to their equal performance as TDs across different conditions. Overall, this suggests that the lack of visuomotor experience prompts the observers to rely on the configural information that they are familiar with, while their ability to pick up the dynamic information remains limited, which makes them more susceptible to the inversion effect of biological motion perception.

The current study is unique in that it addressed the influence of visuomotor experience on the inversion effect of biological motion perception. Our results support previous studies showing that visual and motor experiences are both effective in helping observers use configural and dynamic information to improve biological motion perception. Additionally, they broaden the current understanding of the inversion effect in biological motion perception, demonstrating that the extended visuomotor experience with inverted movements can rectify the inversion effect by remedying the impaired configural and dynamic information. Future studies are warranted to investigate potential changes in visual search patterns when viewing PLDs of inverted movements as the experience of observing and/or performing the inverted movements increases.

## Data Availability

The study reported in this article was not preregistered. The data and code have not been made available on a permanent third-party archive. Requests for the data and code can be sent to the corresponding author.
